# Supplementary material for: Activation of PI3K/Akt Signaling Pathway in Rat Hypothalamus Induced by an Acute Oral Administration of D-Pinitol
Source: Nutrients. 2021 Jun 30;13(7):2268. doi: 10.3390/nu13072268 (PMC8308282; doi:10.3390/nu13072268)

Supplementary material

Supplementary Table S1. Antibodies used for protein analysis by Western blotting.

**Antigen**	**Manufacturing details**	**Dilution**
γ-Adaptin	BD Transduction Laboratories (#610385)	1/2000
Phospho-GSK-3 α/β (Tyr279/Tyr216)	Merck Millipore (#15648)	1/1000
Phospho-GSK-3β (Ser9) [D85E12]	Cell Signalling Technology (#5558)	1/1000
GSK-3β [D5C5Z]	Cell Signalling Technology (#12456)	1/1500
AMPK-α [D63G4]	Cell Signalling Technology (#5832)	1/1000
Phospho-AMPK-α (Thr172) [40H9]	Cell Signalling Technology (#2535)	1/1000
PTEN [D4.3]	Cell Signalling Technology (#9188)	1/1000
PP2Cα	R&D Systems, Inc. (#MAB4150)	1/1000
Phospho-PKAα/β/γ (Thr198)	Santa Cruz Biotechnology, Inc. (#sc-32968)	1/200
PKAα cat (C-20)	Santa Cruz Biotechnology, Inc. (#sc-903)	1/200
Phospho-PI3K-p85 (Tyr607)	Abcam (#182651)	1/1000
PI3K-p85 [19H8]	Cell Signalling Technology (#4257)	1/1000
Phospho-Akt (Ser473)	Cell Signalling Technology (#9271)	1/1000
Akt	Cell Signalling Technology (#9272)	1/1000
Phospho-mTOR (Ser2448)	Cell Signalling Technology (#2971)	1/1000
mTOR	Cell Signalling Technology (#2972)	1/1000
Phospho-GS (Ser641)	Cell Signalling Technology (#3891)	1/1000
Glycogen Synthase (15B1)	Cell Signalling Technology (#3886)	1/1000
Phospho-IRS1 (Ser612) [C15H5]	Cell Signalling Technology (#3015)	1/1000
Phospho-IRS1 (Tyr896) [EP260Y]	Abcam (#ab46800)	1/1000
IRS-1 [D23G12]	Cell Signalling Technology (#3015)	1/1000

Figure S1: Two membranes were used for mTOR, GS, α-Adaptin, Akt and GSK-3β immunoblotting (unedited blots).

Whole membranes 1 and 2. Red Ponceau Staining and individual membranes are shown below. mTOR (1), GS (2), Akt (3) and GSK-3β (4) bands are represented in Figures 4 and 5. All bands were quantified for histograms charts and statistical analysis, although only representative bands were shown in the Figure 4. All proteins were normalized with its respective γ-Adaptin (2).

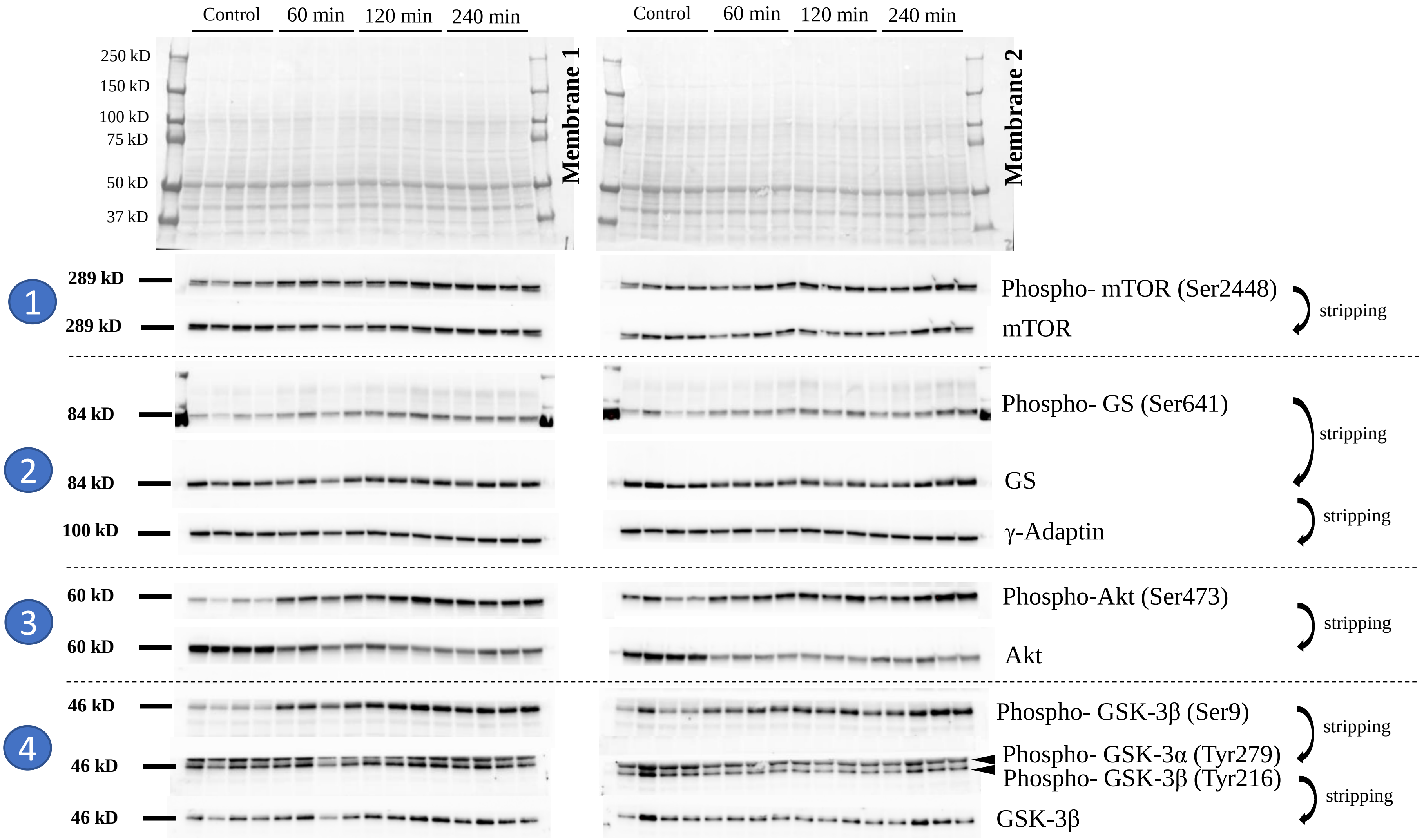

Figure S2: Two membranes were used for IRS1, α-Adaptin, PI3K-p85 and PKAα immunoblotting (unedited blots).

Whole membranes 3 and 4. Red Ponceau Staining and individual membranes are shown below. IRS-1 (1), PI3K-p85 (2) and PKA (3) bands are represented in Figures 4 and 6. All bands were quantified for histograms charts and statistical analysis, although only representative bands were shown in the Figure. All proteins were normalized with its respective γ-Adaptin (1).

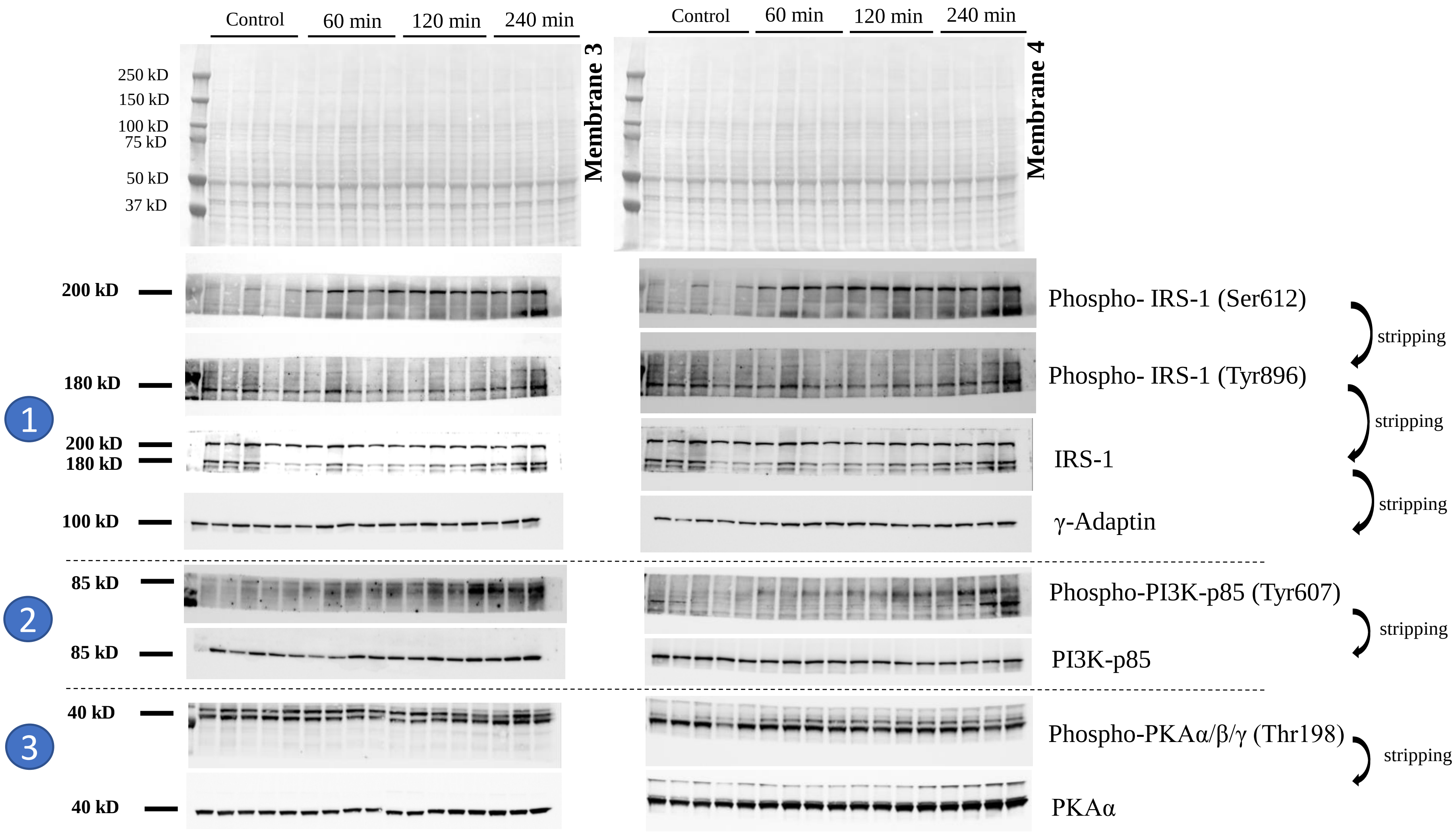

Figure S3: Two membranes were used for α-Adaptin, AMPKα, PTEN and PP2C immunoblotting (unedited blots).

Whole membranes 5 and 6. Red Ponceau Staining and individual membranes are shown below. AMPKα (2) bands are represented in Figure 4. PTEN and PP2C (3) bands are represented in Figure 3. All bands were quantified for histograms charts and statistical analysis, although only representative bands were shown in Figure 6. All proteins were normalized with its respective γ-Adaptin (1).

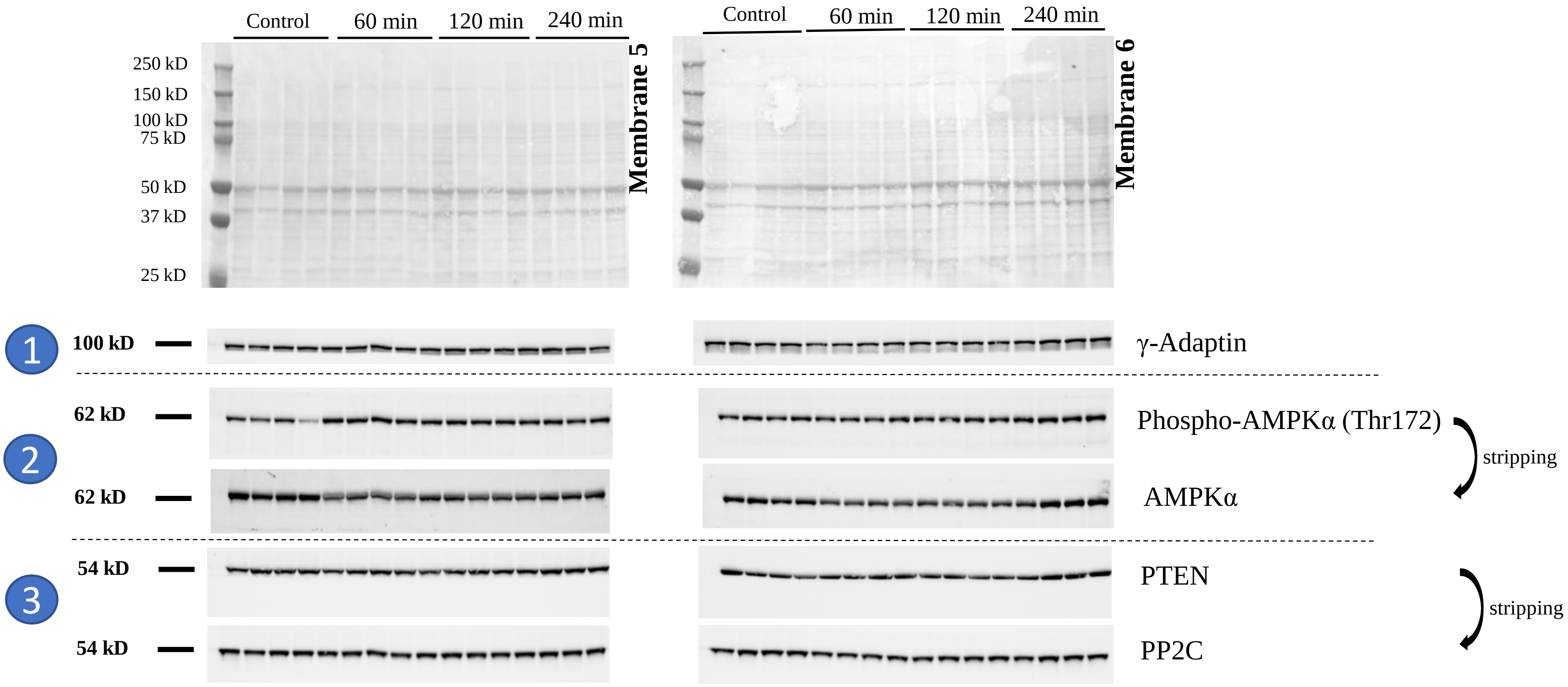

Supplement: Supplementary file 1 [file nutrients-13-02268-s001.zip › nutrients-1267328-supplementary.pdf]
